# The effect of breast cancer surgery on spine alignment: Whole-spine radiograph analysis

**DOI:** 10.1371/journal.pone.0276173

**Published:** 2022-10-14

**Authors:** Kyung Eun Nam, Inah Kim, Hae-Yeon Park, Jong In Lee

**Affiliations:** Department of Rehabilitation Medicine, Seoul St. Mary’s Hospital, College of Medicine, The Catholic University of Korea, Seoul, Republic of Korea; Universidad de Almería, SPAIN

## Abstract

Breast cancer survivors may experience spinal deformity following breast cancer surgery. This study investigated the long-term effects of breast cancer surgery on whole-spine alignment. This retrospective study included 200 patients who underwent breast cancer surgery and ≥2 anteroposterior standing whole-spine X-rays. The curvature of the spine was measured using the Cobb angle; changes in Cobb angle between X-rays were compared among three groups according to breast cancer surgery type. The mean interval between initial and follow-up X-ray was 28.46 ± 13.39 months. The change in Cobb angle was 0.40 ± 1.65 degrees and the absolute value of that change was 1.25 ± 1.15 degrees in all patients with breast cancer. There were no significant differences in angular change among groups according to breast cancer surgery type. Most patients showed minimal changes in spinal alignment after breast cancer surgery. Our findings indicate that breast cancer surgery does not negatively affect spinal alignment.

## Introduction

Breast cancer is the most common cancer among Korean women, with a 4.3% increase in the incidence annually [1, 2]. Because of early detection and more effective treatment, increasing numbers of women have had extended survivals after breast cancer treatment. Thus, there is growing awareness of the long-term effects of breast cancer treatment including surgery (e.g., breast-conserving surgery; mastectomy; and immediate, delayed, or no reconstruction) and adjuvant therapies (e.g., chemotherapy, radiation, or hormone therapy).

Various physiological changes following surgical management (e.g., pain or limited upper limb motion) could trigger an adaptive change in posture [3–5]. Some studies have reported that breast weight influences an individual’s center of gravity, which leads to changes in posture after breast surgery [5–7]. Furthermore, the loss of flexibility and mobility in irradiated skin and subcutaneous tissue can lead to undesired postural changes. There has been considerable interest in postural changes after breast cancer treatment, often analyzed using photogrammetry or Moire topography. However, the results of previous studies have been inconsistent [5, 8, 9]. In addition, current treatments for breast cancer adversely affect bone health through several mechanisms. Aromatase inhibitors cause significant reductions in bone mineral density [10]. Similar effects are also associated with tamoxifen use in premenopausal women [11]. Adjuvant chemotherapy has a substantial impact on bone health due to its induction of premature menopause and its direct effects on bone turnover [12].

In addition, breast cancer survivors are at elevated risk for spinal abnormalities (e.g., scoliosis) because of post-treatment changes in posture and bone health. Other authors [13–19] have evaluated the relationship between breast cancer surgery and spine deformity by means of radiological assessment. However, the findings of these studies are inconsistent, and most studies employed chest radiographs or dual energy X-ray absorptiometry to identify only thoracic or lumbar regions of the spine. Therefore, here, we conducted whole-spine anteroposterior standing radiographic assessment to examine the long-term effects of breast cancer surgery on spinal alignment.

## Materials and methods

### Patient population

**Fig 1:**
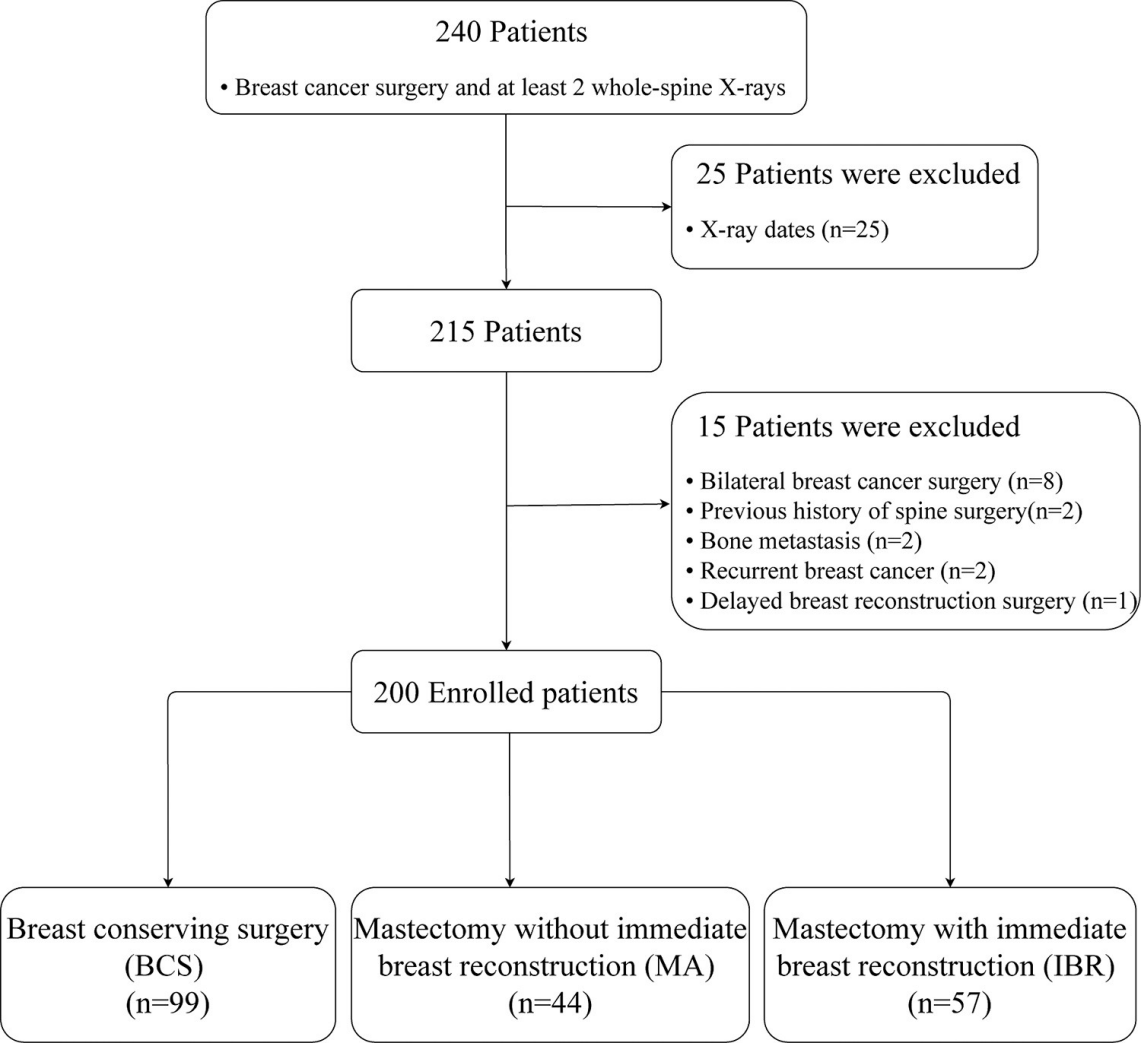
Flow diagram depicting the study’s patient selection process.

This retrospective study was carried out in the Department of Rehabilitation at Seoul St. Mary’s hospital between April 2014 and August 2020. Patients were eligible for the study if they met the following criteria: diagnosed with breast cancer; surgical procedure (e.g., breast-conserving surgery [BCS], mastectomy [MA] alone, or MA with immediate breast reconstruction [IBR]); and ≥2 whole-spine anteroposterior standing X-rays. The first X-ray was performed within 60 days postoperatively and a follow-up X-ray was conducted ≥ 300 days later. Of the 240 patients who underwent breast cancer surgery and had at least 2 whole-spine X-rays, 25 patients were ruled out because their X-ray dates did not fit into the appropriate time frame. This left us with 215 patients who were eligible for participation. Exclusion criteria were bilateral breast cancer surgery, previous history of spine surgery, previous history of treatment (e.g., chemotherapy, radiation, or hormone therapy for other cancers), bone metastasis, recurrent breast cancer, and delayed breast reconstruction surgery. Fifteen patients were excluded because of these criteria; therefore, 200 patients were included in the final analyses. We divided the patients into three groups according to the surgical procedure: BCS, MA alone, and MA with IBR (i.e., IBR group). The flow diagram depicting the patient selection process is outlined in Fig 1. This study followed the STrengthening the Reporting of OBservational studies in Epidemiology (STROBE) guidelines for the reporting observational studies.

### Data collection and definitions

**Fig 2:**
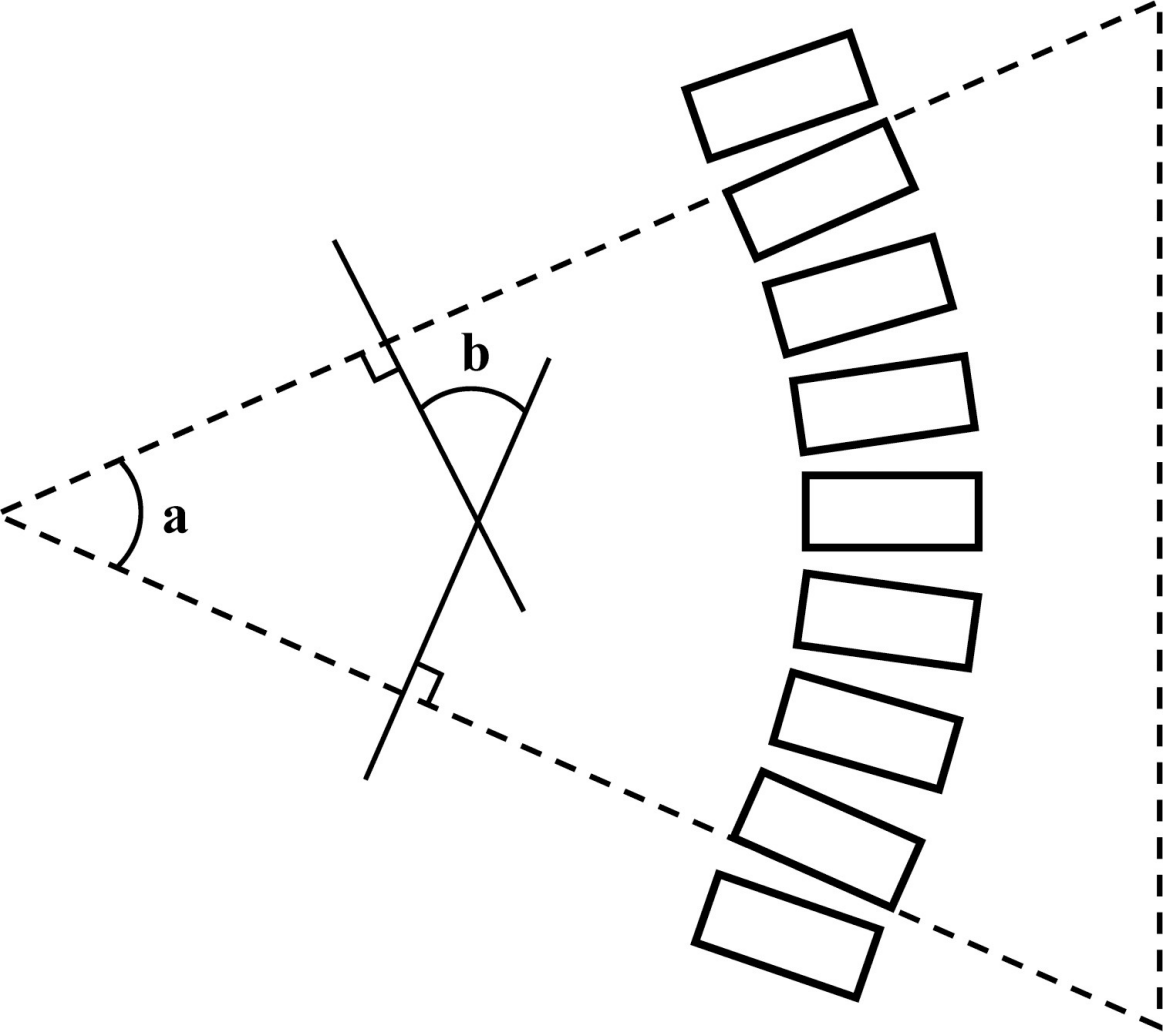
Schematic illustration of Cobb angle measurement.

We examined electronic medical records to collect demographic and clinical information relevant to spinal alignment, as follows: age, body mass index (BMI), presence of osteoporosis, and type of breast cancer treatment (e.g., surgery, chemotherapy, radiation therapy, and hormone therapy). BMI was calculated as weight in kilograms divided by height in meters square (kg/m^2^). Osteoporosis was confirmed via bone mineral density analyses. Spinal curvature was measured using the Cobb angle on whole-spine X-ray. The spinal segment with the greatest curvature was determined and two lines were marked on the film: a line perpendicular to the superior end plate of upper-end vertebrae and a line perpendicular to the inferior end plate of lower-end vertebrae (Fig 2). The same endpoints were used at initial and follow-up curve assessment to reduce measurement error [20–23]. The Cobb angle was automatically calculated using the angle measurement tool provided by the picture archiving and communication system (i.e., the digitized system that stores radiographs and medical records). Scoliosis was defined as ≥10 degrees of curvature. The anatomical site of the apex in the frontal plane was categorized as thoracic (from disc T1–2 to disc T11–12), thoraco-lumbar (from T12 to L1), or lumbar (from disc L1–2) [24]. Angular change was defined by subtracting the Cobb angle in the initial X-ray from the Cobb angle in the follow-up X-ray. A positive angular change indicated an increased spinal curve, while a negative angular change indicated a decreased spinal curve. The absolute value of angular change indicated the extent of change in the Cobb angle, regardless of direction.

Thirty-three radiographs were selected by simple random sampling without replacement to determine the intra- and interobserver variabilities of Cobb measurements. To determine intraobserver variability, clinician 1 (K.E.N) measured the Cobb angle in 33 randomly selected radiographs, with the second set of measurements performed 2 weeks later. To assess interobserver variability, clinician 2 (I.K) who was blinded to the measurements by clinician 1 measured the Cobb angle in the same 33 radiographs and compared the measurements with the first set obtained by clinician 1. And then, clinician 1 examined all radiographic features in our study. Both intra- and interobserver reliabilities were accessed by calculating intraclass correlation coefficients (two-way mixed, random effect model, absolute agreement). Reliabilities were excellent for all comparisons: the intraobserver and interobserver intraclass correlation coefficients were 0.947 and 0.926, respectively. The most commonly cited error in radiographic acquisition or Cobb angle measurement is 5 degrees [20–22, 24–27]. Therefore, we defined a significant progressive angular change as >5 degrees in follow-up X-ray analyses. The Human Research Ethics committee of the Catholic University of Korea approved this retrospective study and waived the requirement for obtaining informed consent.

### Statistical analyses

The results are presented as means and standard deviations (SDs) for quantitative variables, and are summarized as absolute frequencies and percentages for categorical variables. Analysis of variance with *post hoc* Tukey’s honestly significant difference test, chi-square test, or Fisher’s exact test was performed to explore differences among three groups according to the type of surgery. Associations of scoliosis and age categories were analyzed using linear-by-linear association. All statistical tests were conducted using SPSS Statistics for Windows (version 24.0; IBM, Armonk, NY, USA). P-values < 0.05 were considered to indicate statistical significance.

## Results

### Demographic and clinical characteristics

The baseline demographic and clinical characteristics of patients are shown in Table 1. There were differences in age and BMI among the three groups (P = 0.002 and P = 0.023, respectively). *Post hoc* analyses demonstrated that women in the IBR group were younger than women in the BCS and MA groups (P = 0.015 and P = 0.003, respectively). Following adjustment for age, analyses of covariance showed no significant difference in BMI (P = 0.144). The BCS group also included a large proportion of patients who received radiation (P < 0.001), because BCS typically was followed by radiation to eradicate any microscopic residual disease.

**Table 1:** Demographic and clinical characteristics.

Characteristics	Total	BCS	MA	IBR	p-value
	(n = 200)	(n = 99)	(n = 44)	(n = 57)	
Age (years)	49.78 ± 9.60	50.61 ± 9.95	52.48 ± 9.94	46.25 ± 7.67	0.002*
BMI (kg/m^2^)	22.85 ± 2.91	23.22 ± 3.06	23.18 ± 2.77	21.96 ± 2.58	0.023* (0.144^†^)
Surgery side (Left)	97 (48.5%)	47 (47.5%)	27 (61.4%)	23 (40.4%)	0.107
Chemotherapy	152 (76.0%)	72 (72.7%)	35 (79.5%)	45 (78.9%)	0.561
Chemotherapy (n = 154)^‡^	106 (68.8%)	63 (70.0%)	18 (66.7%)	25 (67.6%)	0.931
Radiation	128 (64.0%)	90 (90.9%)	18 (40.9%)	20 (35.1%)	< 0.001*
Hormone therapy	157 (78.5%)	78 (78.8%)	34 (77.3%)	45 (78.9%)	0.975
Osteoporosis	67 (33.5%)	38 (38.4%)	16 (36.4%)	13 (22.8%)	0.126

Data expressed as mean ± SD or number (%).

*p < 0.05 indicates statistical significance.

^†^p-value after performing ANCOVA adjusted for age.

^‡^154 patients were analyzed after exclusion of 46 patients who received neoadjuvant chemotherapy before initial X-ray.

### Spinal alignment in breast cancer survivors

**Fig 3:**
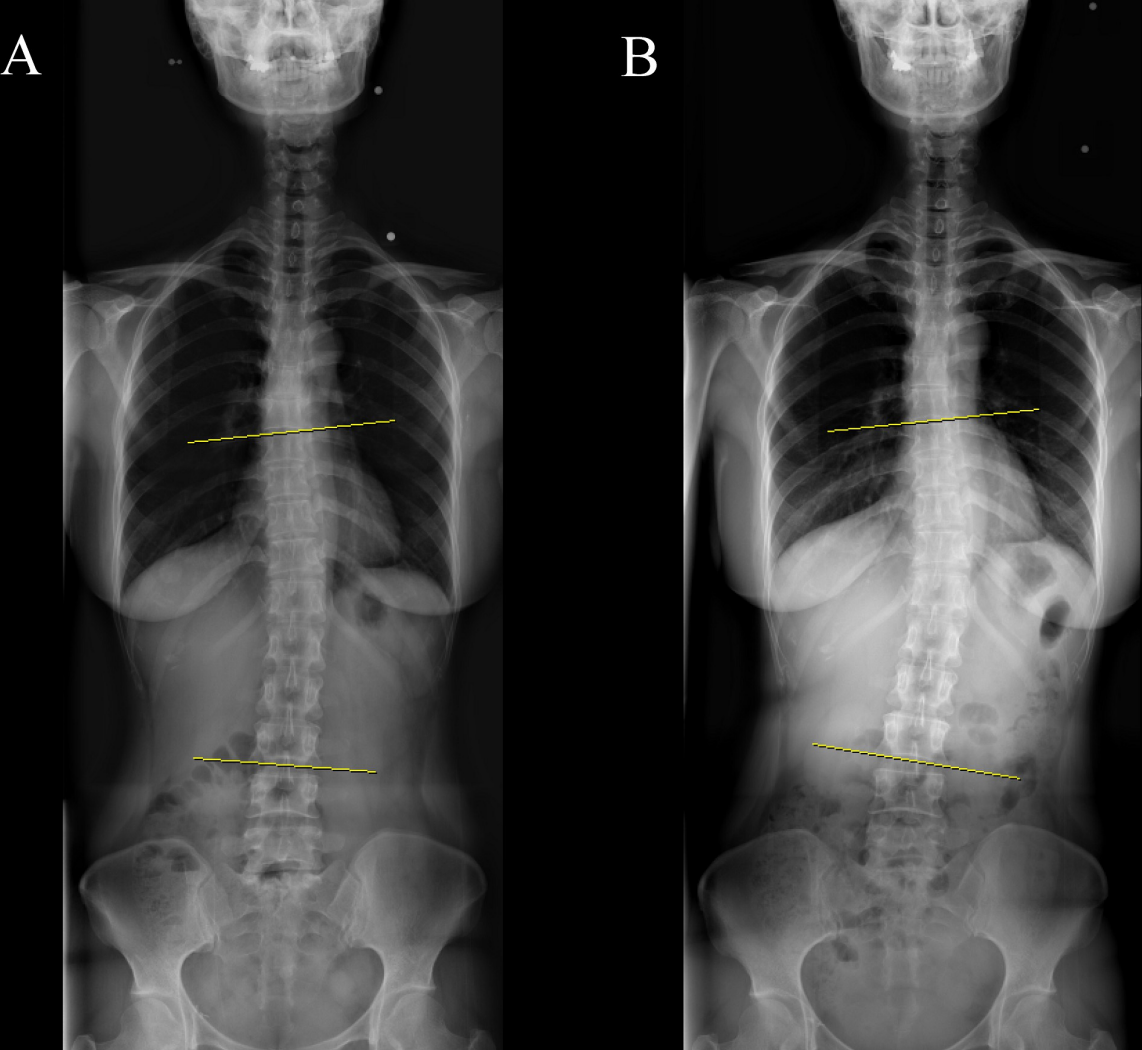
Demonstration of Cobb angle on whole-spine radiograph in one patient with a significant change in spinal curve; (A) Initial radiograph (B) Follow-up radiograph.

At initial radiological assessment, the mean Cobb angle (±SD) was 5.40 ± 4.06 degrees and scoliosis was present in 25 (12.5%) of 200 breast cancer survivors. All but one patient had mild scoliosis (10–20 degrees). There were no significant differences in Cobb angles and proportions of scoliosis among the three surgery groups (Table 2). The proportions of scoliosis according to age category (<40 years, 40–49 years, 50–59 years, and ≥60 years) were 13.8% (4/29), 7.8% (6/77), 9.7% (6/62), and 28.1% (9/32), respectively. Although the proportion of scoliosis did not significantly differ according to age (P = 0.07), the greatest proportion of patients with scoliosis was among patients ≥ 60 years of age. The mean interval between initial and follow-up X-ray was 28.46 ± 13.39 months (median, 26.41 months; interquartile range, 17.28–37.53 months). In follow-up X-rays, the mean change and mean absolute value of the change in Cobb angle were 0.40 ± 1.65 degrees and 1.25 ± 1.15 degrees, respectively. There were no significant differences in angular change or absolute value of angular change among the three groups. However, three patients (1.5%) showed a significant progressive angular change in follow-up X-ray (Fig 3). Only one patient without scoliosis in the initial X-ray was diagnosed with scoliosis in the follow-up X-ray.

**Table 2:** Spinal alignment in breast cancer survivors.

Characteristics	Total	BCS	MA	IBR	p-value
	(n = 200)	(n = 99)	(n = 44)	(n = 57)	
Cobb angle, initial	5.40 ± 4.06	5.22 ± 3.93	5.74 ± 4.62	5.45 ± 3.87	0.771
Cobb angle, follow-up	5.80 ± 4.03	5.74 ± 4.09	5.94 ± 4.14	5.79 ± 3.90	0.963
Interval between X-ray (months)	28.46 ± 13.39	27.08 ± 12.51	31.61 ± 15.51	28.44 ± 12.94	0.175
Angular change	0.40 ± 1.65	0.53 ± 1.72	0.20 ± 1.65	0.34 ± 1.54	0.527
Absolute value of angular change	1.25 ± 1.15	1.26 ± 1.28	1.23 ± 1.11	1.26 ± 0.93	0.985
Scoliosis, initial	25 (12.5%)	13 (13.1%)	6 (13.6%)	6 (10.5%)	0.865
Significant progressive angular change	3 (1.5%)	3 (3.0%)	0 (0.0%)	0 (0.0%)	0.447*
Apex side (Left)	98 (49.0%)	47 (47.5%)	18 (40.9%)	33 (57.9%)	0.218
Anatomical site of apex					0.181
Thoracic	83 (41.5%)	36 (36.4%)	24 (54.5%)	23 (40.4%)	
Thoracolumbar	55 (27.5%)	26 (26.3%)	10 (22.7%)	19 (33.3%)	
Lumbar	62 (31.0%)	37 (37.4%)	10 (22.7%)	15 (26.3%)	

Data expressed as mean ± SD or number (%).

* Fisher Exact test was performed.

## Discussion

This study explored the long-term effects of breast cancer surgery on spinal alignment in patients with breast cancer. There was no association between surgery type and angular change. Furthermore, most patients showed minimal change (i.e., within measurement error) after breast cancer surgery during the mean follow-up period of 28.5 months. Among patients without scoliosis in the initial X-ray, only one patient (in the IBR group) was diagnosed with scoliosis in the follow-up X-ray. In this patient, the initial and follow-up Cobb angles were 8.8 degrees and 11.4 degrees, respectively; this angular change was 2.6 degrees, within the measurement error of 5 degrees. Therefore, the findings in this patient were presumably related to measurement errors rather than new-onset scoliosis. Nonetheless, three patients (all in the BCS group) showed a significant progressive angular change. There was no association between the angular change direction and the surgical side.

Other studies have investigated the relationship between breast cancer surgery and spine deformity by means of radiographic assessment. An analysis of pre- and post-mastectomy chest X-rays in 60 breast cancer survivors reported that spinal alignment shifted to the non-mastectomy side at 1 year postoperatively to balance the weight of the missing breast. A shift in Cobb angle to the mastectomy side was observed in 11 of 53 patients, whereas a statistically significant shift in Cobb angle to the opposite of the mastectomy side was observed in 33 of 53 patients [14]. The average change in Cobb angle was 4.7 after mastectomy in 19 patients with scoliosis. The mass of breast removed significantly correlated with the difference in Cobb angle in nine patients who received unilateral mastectomy. However, laterality of mastectomy was not related to the side of shift in scoliosis curvature [17]. In a study assessing chest radiographs before and after delayed breast reconstruction, the difference was statistically significant and the average change in Cobb angle was 4.3 [16]. Jeong et al. [13] compared the Cobb angle in pre- and post-operative chest X-rays between IBR and MA groups. Without considering curvature change, the difference was -0.593 in the IBR group and 0.335 in the MA group, and considering curvature change, the difference was 2.698 and 3.972 in the IBR and MA group, respectively. They concluded that the IBR group showed significantly smaller changes in postoperative spinal alignment compared with the MA group. Lee et al. [18] also reported that there were significant difference in the Cobb angle between the IBR and MA group when using whole spine (pre- and post-operative Cobb angle; 6.5 and 5.8 in the IBR group, 5.3 and 6.8 in the MA group). However, we should carefully consider the clinical meaning of the results in these previous studies because the value is too small despite statistical significance. By contrast, other previous studies are consistent with our findings. In a study to compare pre-treatment and post- treatment dual energy X-ray absorptiometry scans in 652 breast cancer patients, scoliosis was not associated with surgery, chemotherapy, hormone therapy, BMI, or bone mineral density [15]. Oh et al. [19] reported that most patients did not have a clinically relevant spinal deformity prior to breast reconstruction, which was performed after an average 5 years from mastectomy.

Some studies have investigated the effects of breast cancer treatment on posture by means of photogrammetric assessment. Rostkowska et al. [5] reported that women with MA have a higher scapula on the surgery side. Moreover, women who undergo surgery at an older age more frequently exhibit trunk deviation to the right, along with backward movement of the right side of the pelvis. Recent surgery is associated with forward trunk leaning, while surgery many years prior is associated with backward trunk leaning. Glowacka et al. [9] also demonstrated that the shoulder on the surgery side is lifted and the contralateral shoulder is lowered in patients who undergo BCS or MA, until 1 year postoperatively. The magnitude of this difference is greater in patients who undergo MA. However, that study did not report postoperative trunk imbalance. Peres et al. [8] also compared body postures at 1 to 5 years postoperatively between women who had undergone MA and women who had undergone IBR. Women who had undergone MA showed greater asymmetry between the acromion and greater trochanter of the femur, indicating trunk rotation. However, there were no differences between the two groups with respect to alignment of the head, shoulders, scapula, or pelvis. Overall, the results of previous studies regarding the relationship between breast cancer treatment and postural change have been inconsistent and the mechanism underlying postoperative posture changes is unclear. These discrepancies could arise from differences in the included patients or assessment method.

There were a few limitations to this study. First, we could not control the use of additional bras to cover the missing breast in the MA group, because of the retrospective study design. Among 44 patients in the MA group, 18 did not use an extra bra while 16 did occasionally; we did not find information regarding the use of an extra bra in the remaining 10 patients. However, there were no significant progressive angular changes in the MA group. Second, only three patients showed a significant progressive angular change, which precluded analyses of risk factors regarding significant angular change. Further studies with additional patients would clarify the risk factors for significant angular change. Third, we defined the initial assessment as X-rays performed within 60 days postoperatively. Thus, we could not identify short-term changes that occurred before the initial postoperative X-ray. However, there were no differences in the initial Cobb angles and proportions of scoliosis among the three groups. Moreover, we included patients with an interval time of ≥300 days between X-rays. Hence, short-term postoperative changes had minimal effects on the overall outcome of our study, which focused on long-term effects. Fourth, this study did not perform double assessments for all of the radiographs. To mitigate this limitation, we assessed intra- and interobserver variabilities and reliabilities were excellent. Fifth, patients in our study were not compared to a gender and age-matched control population without breast cancer surgery. Finally, we could not determine complex three-dimensional deformity of the spine, because we used Cobb angle measurement from whole-spine X-rays. However, Cobb angle measurement remains the main standard for diagnosis, monitoring, therapeutic planning, and epidemiologic analyses of patients with scoliosis [24, 25]. Despite these limitations due to the retrospective design, the results of this study are meaningful because they reflect the realistic effects of breast cancer treatment on spinal alignment in real-world clinical practice.

## Conclusion

We found no association between surgery type and changes in spinal alignment. Furthermore, there were minimal changes in spinal alignment after breast cancer surgery, regardless of surgery type. These results suggest that breast cancer surgery does not negatively influence spinal alignment.

## Supporting information

S1 File Dataset. (SAV)
